# The clinical development of antibody-drug conjugates for non-small cell lung cancer therapy

**DOI:** 10.3389/fimmu.2023.1335252

**Published:** 2023-12-11

**Authors:** Xinlin Liu, Junwen Deng, Renshuai Zhang, Jiyao Xing, Yudong Wu, Wujun Chen, Bing Liang, Dongming Xing, Jiazhen Xu, Miao Zhang

**Affiliations:** ^1^The Affiliated Hospital of Qingdao University, Qingdao University, Qingdao, China; ^2^Qingdao Cancer Institute, Qingdao, China; ^3^School of Life Sciences, Tsinghua University, Beijing, China

**Keywords:** NSCLC, antibody-drug conjugate, targeted therapy, clinical outcome, mechanisms of action

## Abstract

Despite the emergence of molecular targeted therapy and immune checkpoint inhibitors as standard first-line treatments for non-small cell lung cancer (NSCLC), their efficacy in some patients is limited by intrinsic and acquired resistance. Antibody-drug conjugates (ADCs), a revolutionary class of antitumor drugs, have displayed promising clinical outcomes in cancer treatment. In 2022, trastuzumab deruxtecan (Enhertu) was approved for treating HER2-mutated NSCLC, thereby underscoring the clinical value of ADCs in NSCLC treatment strategies. An increasing number of ADCs, focusing on NSCLC, are undergoing clinical trials, potentially positioning them as future treatment options. In this review, we encapsulate recent advancements in the clinical research of novel ADCs for treating NSCLC. Subsequently, we discuss the mechanisms of action, clinical efficacy, and associated limitations of these ADCs.

## 1 Introduction

Lung cancer, known as the most common thoracic malignancy, is the leading cause of cancer-related deaths worldwide, revealing a 5-year survival rate of only 10%-20% (1). NSCLC accounts for approximately 85% of lung cancer cases, establishing it as the dominant subtype (2). In recent years, the introduction of targeted therapies and immunotherapy has significantly reshaped the treatment landscape for NSCLC. In 2003, the approval of gefitinib by the Food and Drug Administration (FDA), as the first molecular targeted drug for NSCLC treatment, led the way for the development of potent inhibitors such as EGFR, ALK, RET, and KRAS (3, 4). Nonetheless, a marginal section of patients (25%) benefit from these targeted therapies, whilst drug resistance remains a challenge (5). For most patients with driver-gene-negative NSCLC, immune checkpoint inhibitor (ICI) has surpassed combination chemotherapy as the primary approach (3). However, the effectiveness of ICIs in treating metastatic NSCLC patients remains underwhelming, with a median overall survival (mOS) of less than 3 years. Overcoming drug resistance presents continual obstacles (6, 7). ADCs, an emerging class of antineoplastic drugs, mainly encompass three components: antibody, linker, and cytotoxic payload. This therapeutic approach conveys anti-tumor effects via targeted delivery of cytotoxic drugs into tumor cells, earning it the nickname of a ‘magic bullet’ (8). Currently, ADC development is advancing rapidly. To date, the FDA has approved 12 ADCs for tumor treatment, with 9 of them receiving approval since 2017 (9). Importantly, trastuzumab deruxtecan is the only ADC used to treat HER2-mutated NSCLC, signifying an innovative approach to using ADCs in targeted therapy for NSCLC (10). In this review, we summarize recent advancements in the research of ADCs for the treatment of NSCLC, with a focus on aspects including the mechanisms of action, clinical efficacy, and limitations.

## 2 Trop2-targeted ADCs

Trophoblast cell surface antigen 2 (Trop2), a type I cell surface glycoprotein, shows limited expression in normal tissues but over-expression in various types of tumors, including breast cancer, NSCLC, pancreatic cancer, and other tumors. In NSCLC, over-expressed Trop2 is associated with lymph node metastasis and poor OS (11). There have been several clinical trials to evaluate the therapeutic potential of Trop2-targeted ADCs in NSCLC.

### 2.1 Sacituzumab govitecan (Trodelvy)

Sacituzumab govitecan is a Trop2-targeted ADC composed of hRS7, an anti-Trop2 monoclonal antibody, connecting to the irinotecan metabolite (SN-38) via a cleavable CL2A carbonate linker with a DAR of 7.6. Preclinical studies demonstrated that sacituzumab govitecan can selectively bind to Trop2^+^ tumor cells, causing double-stranded DNA breaks and tumor cell death by topoisomerase I and bystander effect. Sacituzumab govitecan exhibits potent antitumor effects *in vitro* and *in vivo*. Furthermore, it is well tolerated in monkeys at clinically relevant doses (12, 13). Sacituzumab govitecan is currently the only Trop2-targeted ADC approved by the FDA for the treatment of metastatic triple-negative breast cancer (mTNBC) patients who have received at least two prior therapies for metastatic disease. In a first-in-human (FIH) 1/2 clinical trial of sacituzumab govitecan (NCT01631552), an ORR of 19% was observed among 47 evaluated NSCLC patients. The 8 and 10 mg/kg doses every 21 days were selected as the recommended phase 2 dose (RP2D). Notably, neutropenia was reported by 43% of the patients. Grade 3 or higher AEs were experienced by 5% of the patients, including diarrhea (7%), nausea (7%), fatigue (6%), and neutropenia (28%) (14, 15). Currently, there are several ongoing clinical trials aimed at evaluating the clinical activity of sacituzumab govitecan as a single agent or in combination with other antitumor agents for patients with NSCLC. In a trial (NCT05186974) with sacituzumab govitecan combined with first-line therapy (pembrolizumab or platinum agent), preliminary data demonstrated an ORR of 56% among 61 patients with advanced or metastatic NSCLC that receiving treatment of combination of sacituzumab govitecan and pembrolizumab (16). In addition, three clinical trials (NCT06055465, NCT05089734, and NCT05609968) in patients with refractory or advanced metastatic NSCLC are ongoing.

### 2.2 Datopotamab deruxtecan

Datopotamab deruxtecan, jointly developed by Daiichi Sankyo and AstraZeneca, aims at treating metastatic breast cancer and metastatic NSCLC. It consists of an anti-Trop2 monoclonal antibody, an enzymatically cleavable tetrapeptide linker, and the exatecan derivative (DXd) (17). Unlike sacituzumab govitecan and other deruxtecan-containing ADCs, datopotamab deruxtecan has a lower DAR. This design is based on preclinical studies suggesting that datopotamab deruxtecan with a DAR of 4 offers enhanced tolerability and a broader therapeutic window in cynomolgus monkeys, in contrast to those with higher DARs. The mechanism of action (MOA) of datopotamab deruxtecan includes inhibition of DNA topoisomerase I and bystander effects (17). Datopotamab deruxtecan is currently undergoing phase 3 clinical trials. The safety, tolerability, and preliminary efficacy of datopotamab deruxtecan have been evaluated in a phase 1 clinical trial (NCT03401385) involving patients with advanced solid tumors. A total of 180 patients with NSCLC received datopotamab deruxtecan, and the treatment of datopotamab deruxtecan showed both antitumor activity and safety. The blinded independent central review determined the ORR as follows: 4 mg/kg - 24% (12/50), 6 mg/kg - 26% (13/50), and 8 mg/kg - 24% (19/80). Among the patients, 47% experienced grade 3 or worse TEAEs, with the most common being nausea, stomatitis, alopecia, and fatigue (18). Notably, the 6 mg/kg dose demonstrated better tolerability, greater effectiveness, and lower AEs, establishing it as the recommended dosage for future development. As a result, it has been recommended for use in subsequent clinical trials. Based on the notable clinical value exhibited by datopotamab deruxtecan in this trial, further evaluation is being conducted through the TROPION-LUNG program. This extensive clinical development initiative aims to assess the effectiveness and safety of datopotamab deruxtecan, either as a monotherapy or in combination with other antitumor agents, specifically targeting Trop2^+^ NSCLC (11). TROPION-Lung01 (NCT04656652) is a phase 3 clinical trial that assesses the effectiveness and safety of datopotamab deruxtecan when compared to docetaxel (DTX) in individuals with advanced or metastatic NSCLC who have received previous treatment. The study enrolled a total of 604 patients, with 299 in the datopotamab deruxtecan group and 305 in the DTX group. The observed ORR was significantly higher in the datopotamab deruxtecan group (26.4%) compared to the DTX group (12.8%). It is noteworthy that the datopotamab deruxtecan group had a higher occurrence of grade ≥ 3 treatment-related interstitial lung disease (ILD) compared to the DTX group, with rates of 3.4% and 1.4% respectively. Stomatitis (49.2%) and nausea (37%) were the most frequently reported TEAEs in the datopotamab deruxtecan group (19). TROPION-Lung02 (NCT04526691) is a phase 1 clinical trial evaluating the efficacy of datopotamab deruxtecan plus pembrolizumab in patients with advanced NSCLC with or without chemotherapy. As of April 2023, a 38% ORR was observed in 61 patients with advanced NSCLC who received datopotamab deruxtecan plus pembrolizumab treatment. Additionally, the ORR for patients receiving platinum-based chemotherapy in combination with datopotamab deruxtecan and pembrolizumab (n = 71) was 49%. In terms of safety, grade 3 or higher TEAEs occurred in 53% and 76% of patients in the two groups who received doublet or combination chemotherapy, respectively (20). The TROPION-Lung05 (NCT04484142), phase 2 clinical trial, aims to examine both the effectiveness and safety of datopotamab deruxtecan in individuals with advanced or metastatic NSCLC who have actionable genomic alterations. Out of 137 patients who received datopotamab deruxtecan treatment, 56.9% had EGFR mutants. As of December 2022, the observed ORR was 35.8%. Patients with EGFR mutants showed a comparable response, with an ORR of 43.6%. The most common grade ≥ 3 TEAEs were stomatitis (9.5%), anemia (5.8%), and increased amylase (5.8%) (21). Other TROPION-Lung clinical trials (TROPION-Lung07 and TROPION-Lung08) are ongoing.

### 2.3 SKB264

In SKB264, an anti-Trop2 antibody (hRS7) is conjugated to a topoisomerase I inhibitor (KL610023, belotecan-derived) via an sulfonyl pyrimidine-CL2A-carbonate linker, resulting in a DAR of 7.4 (22). SKB264 shares a similar MOA with sacituzumab govitecan and datopotamab deruxtecan, exerting anti-tumor effects through the inhibition of topoisomerase I and bystander effects. Preclinical studies have demonstrated the remarkable efficacy of SKB264 in the nonclinical Trop2-expressing patient-derived xenografts (PDX) models, with an acceptable safety profile and an excellent therapeutic window in animal studies (23). A phase 1/2 clinical study (NCT04152499) is evaluating the clinical activity of SKB264 in patients with solid tumors who have shown resistance to standard therapies. In the phase 2 expansion cohort, an ORR of 44% was observed among the 39 NSCLC patients. It is worth noting that patients with tyrosine kinase inhibitors (TKIs)-resistant EGFR mutants appear to be more responsive to SKB264 than patients with EGFR WT. Among the EGFR WT group, the ORR was 26% (5/19), while the TKI-resistant EGFR mutant group demonstrated an ORR of 60% (12/20). The most frequently observed grade ≥ 3 TEAEs, experienced by at least 5% of patients, consisted of neutrophil count decreased (32.6%), anemia (30.2%), white blood cell count decreased (23.3%), stomatitis (9.3%), rash (7.0%), and lymphocyte count decreased (7.0%) (24). Grade 4 TEAEs occurred only for neutropenia and white blood cell count decreased. According to the positive result of SKB264 in the EGFR mutant group, a phase 3 clinical trial (NCT05870319) in patients with EGFR-mutated NSCLC has been initiated to further determine the clinical activity of SKB264. In addition, two clinical studies (NCT05816252 and NCT05351788) aiming to investigate SKB264 in patients with advanced NSCLC are ongoing.

## 3 HER2-targeted ADCs

Human epidermal growth factor receptor 2 (HER2), a member of the epidermal growth factor receptor family, can initiate various oncogenic signaling pathways (MAPK, PI3K, AKT, and PKC), provoking abnormal cell proliferation and encouraging tumorigenesis (25). HER2 is overexpressed across various tumor types. The remarkable success of HER2-targeted therapies in treating HER2^+^ breast cancer encourages us to explore their potential beyond breast cancer. Recent studies demonstrated that NSCLC, closely correlated with abnormal HER2, may also exhibit potential for suitability towards HER2-targeted agents (26, 27). Moreover, receptor ubiquitination and internalization induced by HER2 amplification or mutant offer a mechanistic foundation for employing HER2-targeted ADCs in the treatment of NSCLC (28).

### 3.1 Trastuzumab emtansine (Kadcyla)

Trastuzumab emtansine, the first HER2-targeted ADC to be developed, consists of trastuzumab, connecting to the microtubule inhibitor emtansine (DM1) via a non-cleavable linker with a DAR of 3.5 (29). FDA has approved trastuzumab emtansine in treating advanced HER2^+^ breast cancer patients who have previously received trastuzumab and taxane therapies, either as monotherapy or in combination. Recent encouraging clinical results have demonstrated the therapeutic potential of trastuzumab emtansine for the treatment of HER2^+^ NSCLC. In a phase 2 clinical study (NCT02675829) involving 49 patients with HER2 amplification or mutant, trastuzumab emtansine showed an ORR of 51% (25/49) and a median progression-free survival (mPFS) of 5 months with good tolerability. According to these positive results, the NCCN has recommended trastuzumab emtansine as the only preferred 2L treatment option for metastatic HER2-mutated NSCLC (28). However, another phase 2 clinical trial (UMI000019446) was terminated because of the limited efficacy of trastuzumab emtansine in patients with HER2^+^ relapsed NSCLC. In this setting, 15 patients (33% with IHC3^+^, 20% with IHC2^+^, and 47% with exon 20 mutant) received trastuzumab emtansine with an RP2D of 3.6 mg/kg every three weeks. Only one patient with mutant, accounting for 6.7% (1/15), achieved PR (30). In a phase 2 trial (NCT02289833), trastuzumab emtansine was administered to 49 patients with HER2^+^ NSCLC (29 with IHC2^+^ and 20 with IHC3^+^). No treatment responses were observed in the IHC2^+^ group, whereas 4 patients in the IHC3^+^ cohort achieved partial response (PR). This result suggested the selective activity of trastuzumab emtansine in NSCLC with a high HER2 level (31). In summary, not all patients with HER2^+^ NSCLC benefit from trastuzumab emtansine treatment. The mechanisms of treatment resistance include the disruption of trastuzumab-mediated effects, abnormal changes in trafficking/metabolism, and impairment of lysine-MCC-DM-1-mediated cytotoxicity.

### 3.2 Trastuzumab deruxtecan (Enhertu)

Trastuzumab deruxtecan, an anti-HER2 ADC developed by Daiichi Sankyo and AstraZeneca, is composed of trastuzumab linked with topoisomerase I inhibitor (Deruxtecan, DXd) via a hydrolyzable tetrapeptide linker with a DAR of 8 (32). Owing to its highly permeable payload, trastuzumab deruxtecan exhibits a pronounced bystander effect, which allows it to keep potent antitumor activity even in HER2^low^ tumor cells (33). In 2022, the FDA granted accelerated approval to trastuzumab deruxtecan for HER2-mutated NSCLC. This approval is derived from the significant therapeutic effect observed in the phase 2 DESTINY-Lung01 trial (NCT03505710). The trial involved 91 patients with advanced HER2^+^ NSCLC, who received RP2D at 6.4mg/kg every three weeks and not respond to previous treatments, which included 42 patients with HER2 mutant and 49 patients with HER2 overexpression. For the HER2 mutant group, one achieved complete response (CR) (2.4%), and 25 achieved PR (59.5%) totaling an ORR of 61.9% (34). Trastuzumab deruxtecan showcased an advantageous anti-tumor effect in this category, denoted as significant. In contrast, the cohort showing HER2 overexpression exhibited substantial toxicity and relatively reduced efficacy, with an ORR of 24.5%. Only one of these 49 patients reached CR, with 11 achieving PR, and the mPFS of 5.4 months (35). Among the 91 patients treated with trastuzumab deruxtecan, 88 reported AEs, including 42 instances of grade 3 and above AEs and two documented fatalities. Nausea, neutropenia, and ILD were the most frequent AEs. Drug-related ILD was reported at 26% across all grades and 6.6% for grade 3 and above, respectively (36). Presently, six active clinical trials involving trastuzumab deruxtecan are underway for NSCLC patients, including NCT05048797, a phase 3 study designed to assess both the efficacy and safety of trastuzumab deruxtecan in NSCLC patients with HER2 exon 19 or 20 mutant.

### 3.3 Trastuzumab botidotin

Trastuzumab botidotin is designed for the treatment of HER2^+^ solid malignancies. Its synthesis involves conjugating trastuzumab to duostatin-5 (an auristatin derivative) using a protease-cleavable linker with a DAR of 2. In preclinical studies, trastuzumab botidotin demonstrated better tumor growth inhibition than trastuzumab emtansine at a dose of 3 mg/kg in PDX models (37). The initial clinical trial (NCT03602079) of trastuzumab botidotin incorporated 35 patients suffering from locally advanced or metastatic solid tumors, including NSCLC, that were HER2^+^ or HER2^-^amplified. Preliminary data demonstrated promising anticancer efficacy at the dosages of 3.6 mg/kg and 4.8 mg/kg. Among 27 evaluable patients, which included NSCLC cases, 7 showed PR, contributing an ORR of 36%. The most frequently observed TEAEs included keratitis, decreased appetite, and dry eye, alongside blurred vision and others. Ocular toxicity was particularly prominent. The onset rate of ocular toxicity marked 80% in the 3.6 mg/kg therapy group, whereas the 4.8 mg/kg group reported an 83% incidence rate (38). Three patients exhibited more severe whorl pattern epitheliopathy, indicating limbal stem cell deficiency (LSCD), which necessitated the cessation of treatment (39). Ocular toxicity was also reported in another clinical trial of trastuzumab botidotin (CTR20181301). A total of 81 patients with advanced solid tumors received trastuzumab botidotin treatment, resulting in objective partial tumor responses observed in 43 patients, reflecting an ORR of 53% (40). The most frequent TEAEs, at grades 3 or above, comprised of corneal epitheliopathy (30.9%), blurred vision (18.5%), dry eyes (7.4%), and peripheral sensory neuropathy (6.2%) (41).

### 3.4 SHR-A1811

SHR-A1811 is a HER2-targeted ADC composed of trastuzumab via a cleavable linker and a novel topoisomerase I inhibitor payload (SHR9265, exatecan derivative), with a DAR of 5.7. In preclinical studies, SHR-A1811 showed growth inhibition and antitumor activity in breast cancer and gastric cancer cell lines with different HER2 expression levels (high, medium, and low). Moreover, treated cynomolgus monkeys did not exhibit any deaths or lung injuries within 42 days, indicating a good safety profile (42). NCT04818333 is a phase 1/2 trial evaluating the clinical activity of SHR-A1811 in patients with advanced HER2-mutated NSCLC. A total of 50 patients were enrolled, all of whom had received prior treatment including HER2-targeted TKIs (66%), ICI (68%), and anti-angiogenic drugs (78%). Overall, the ORR was 40%. All patients experienced TEAEs. Grade ≥ 3 TEAEs were observed in 42% of patients, with the most common being decreased neutrophil count (30%), white blood cell count decreased (20%), anemia (16%), and thrombocytopenia (12%). Among the patients, nine (18%) experienced severe AEs that might be associated with SHR-A1811. Two patients had to discontinue treatment due to AEs, and one patient died from treatment-related ILD (43). Additionally, NCT05482568 is an ongoing phase 1/2 clinical trial recruiting patients with advanced NSCLC. It aims to assess the effectiveness of SHR-A1811 when used in combination with either pyrotinib or SHR-1316.

### 3.5 XMT-1522

XMT-1522 is an anti-HER2 ADC made up of the monoclonal antibody HT-19 conjugated with the AF-HPA (auristatin-derivative) payload. It utilizes a cysteine linkage containing biodegradable hydrophilic polymer, with a DAR of 12. AF-HPA and its intracellular metabolite auristatin F (AF) are potent tubulin polymerization inhibitors used to kill tumor cells (44). In preclinical studies, XMT-1522 demonstrated antitumor activity in trastuzumab emtansine-resistant HER2^+^ breast cancer and gastric cancer cell lines as well as trastuzumab emtansine-resistant PDX models (45). In the primary phase 1 clinical trial (NCT02952729) of XMT-1522, a cohort of 19 participants was enrolled, including individuals with HER2^+^ NSCLC. Administered doses of 16 or 21.3 mg/m^2^ led to one patient experiencing PR and four others achieved stable disease (SD), thus yielding a disease control rate (DCR) of 83% (5/6) (46). However, the further development of XMT-1522 was terminated due to a grade 5 TEAEs, resulting in a patient’s death at dose level 7 (47).

## 4 HER3-targeted ADCs

As a member of the EGFR family, human epidermal growth factor receptor 3 (HER3) is found to be abnormally expressed in various malignancies, including NSCLC (48). It triggers the phosphorylation of receptor tyrosine residues by forming homodimers or heterodimers with other EGFR members, thereby activating multiple signaling pathways such as PI3K/AKT and MAPK, leading ultimately to oncogenesis (49). Furthermore, HER3 plays a crucial role in resisting EGFR TKIs and HER2-targeted antibodies (50, 51). This underlines HER3’s potential as a promising therapeutic target for ADC.

### 4.1 Patritumab deruxtecan

Patritumab deruxtecan is an ADC formed by conjugating a humanized anti-HER3 monoclonal antibody (patritumab) to a topoisomerase I inhibitor payload (DXd, exatecan derivative) via a cleavable tetrapeptide linker, with DAR of 4 (52). Preclinical studies indicated that patritumab deruxtecan exhibited robust anti-tumor efficacy in the PDX model overexpressing HER3 via DXd-mediated DNA damage and apoptosis without significant safety concerns (52). In the phase 1 clinical trial (NCT03260491), patritumab deruxtecan demonstrated significant clinical efficacy, which it granted breakthrough therapy designation by the FDA for the treatment of patients with metastatic or locally advanced, EGFR-mutated NSCLC. This trial involved patients diagnosed with locally advanced or metastatic EGFR-driven NSCLC, who had previously undergone treatment with EGFR TKI and platinum-based chemotherapy. Of the 57 patients receiving patritumab deruxtecan, the ORR was 39%, with a mPFS of 8.2 months, and a median duration of response (mDOR) of 6.9 months. Intriguingly, responsiveness was also observed in patients exhibiting resistance to EGFR TKI. It’s noteworthy that nearly all patients reported TEAEs (96%), with 74% experiencing TEAEs of grade 3 or higher. ILD was observed in 5 patients, one of which was of grade 3 (53). Given the encouraging findings of the U31402-A-U102 investigation, the HERTHENA-Lung program was initiated to more thoroughly assess the safety and effectiveness of patritumab deruxtecan in patients bearing EGFR-mutated NSCLC. HERTHENA-Lung01 is a Phase 2 clinical trial (NCT04619004) aimed at evaluating the anti-tumor activity of patritumab deruxtecan in subjects with metastatic or locally advanced NSCLC and an activating EGFR mutant (exon 19 deletion or L858R). As of May 2023, a total of 225 patients have received patritumab deruxtecan treatment, with an ORR of 29.8%, mDOR of 6.4 months, mPFS of 5.5 months, and mOS of 11.9 months. Efficacy has been observed in patients with different HER3 expression levels, different EGFR TKI resistance mechanisms, and those with brain metastases (54). Additionally, the HERTHENA-Lung02 trial (NCT05338970), a Phase 3 trial, is currently ongoing in EGFR-mutated NSCLC patients who have progressed on an EGFR TKI (55).

## 5 c-MET-targeted ADCs

c-MET (Mesenchymal-epithelial transition factor), also known as hepatocyte growth factor receptor, is a receptor tyrosine-protein kinase encoded by the MET gene. Upon ligand binding, c-MET can initiate several signaling pathways including PI3K/AKT and MAPK. These pathways are associated with tumor cellular processes such as proliferation, migration, and invasion (56). In NSCLC, variant forms of c-MET have been observed, including mutant, amplifications, and overexpression (57–59). Notably, a correlation has been established between c-MET amplification and resistance to multiple TKIs (60). Consequently, c-MET has been identified as a promising target in ADC development.

### 5.1 Telisotuzumab vedotin

Telisotuzumab vedotin is a novel ADC produced by conjugating a humanized anti-c-MET monoclonal antibody (ABT-700) to a microtubule inhibitor (MMAE) via a cleavable linker and has a DAR of 3.1 (61). In preclinical studies, telisotuzumab vedotin demonstrated significant tumor growth inhibition and regression in cell lines and PDX models with c-MET overexpression or MET amplification, by targeted delivery of toxins (61). The FIH clinical trial of telisotuzumab vedotin (NCT02099058) was performed on patients with advanced solid tumors showing c-MET overexpression, aiming to evaluate the drug’s safety, tolerability, pharmacokinetics, and maximum tolerable dose. Notably, the results revealed that the response was confined to NSCLC patients. Among the 16 c-MET^+^ NSCLC patients treated with telisotuzumab vedotin, 3 demonstrated a PR, mPFS of 5.7 months, and mDOR of 4.8 months. The RP2D was established at 2.7 mg/kg every 21 days (62). In the following phase 1b trial, the combined efficacy of telisotuzumab vedotin and erlotinib was assessed. The trial involved 42 patients, yielding an overall ORR of 30.6%, and a mPFS of 5.9 months. Among the patients with EGFR mutant (n = 28), the ORR was 32.1% (63). However, in a different phase 2 clinical trial (NCT03539536), telisotuzumab vedotin showed limited efficacy in patients with EGFR mutant. In this trial, telisotuzumab vedotin was used as monotherapy. The ORR was 35.1% for the EGFR WT cohort, while the EGFR mutant cohort had an ORR of 13.3% (64). Overall, for the treatment of c-MET^+^ NSCLC patients with EGFR mutant, the combination of telisotuzumab vedotin and erlotinib might prove more beneficial. Furthermore, the occurrence of TEAEs should be given due attention. A phase 2 clinical trial (NCT03574753) in patients with c-MET^+^ NSCLC, recording an ORR of 9% (2/23), along with 3 fatal grade 5 pulmonary TEAEs (two instances of pneumonia and one of bronchopulmonary hemorrhage) (65). In addition, multiple telisotuzumab vedotin clinical trials (NCT05513703, NCT04928846) are currently underway for patients with advanced/metastatic NSCLC (66, 67).

## 6 EGFR-targeted ADCs

Epidermal growth factor receptor (EGFR), a member of the EGFR family, promotes tumor cell proliferation by activating downstream PI3K/AKT, MAPK, and JAK/STAT signaling pathways. Many malignant tumors, including NSCLC, have been found to carry mutants and amplifications in the EGFR gene (68). Presently, therapy targeting EGFR has been sanctioned as the first-line standard for NSCLC with EGFR mutant (69). Nonetheless, the vast preponderance of therapies targeting EGFR unavoidably engenders resistance, which necessitates the extension of EGFR-targeted therapies, with ADC emerging as a potential candidate (70).

### 6.1 MRG003

MRG003, an EGFR-targeted ADC, consists of an anti-EGFR monoclonal antibody conjugated to a microtubule-disrupting compound, MMAE (71). The safety and antitumor activity of MRG003 patients with either advanced or metastatic solid malignancies were evaluated in a phase 1 clinical trial (NCT04868344). Of 61 patients, 9 (14.7%) achieved PR and 17 (27.8%) documented SD. The recommended dose was determined as 2.5 mg/kg. TEAEs occurred in 89% of the participants, with the majority experiencing grade 1 or 2 AEs. 19 patients (31%) reported grade 3 or higher TEAEs, including hyponatremia, leukocytopenia, neutropenia, elevated aspartate aminotransferase levels, and febrile neutropenia (71). In conclusion, MRG003 administration demonstrated therapeutic potential in patients with EGFR^+^ solid malignancies. Furthermore, a phase 2 study (NCT04838548) examining the efficacy and safety of MRG003 in patients with EGFR^+^ advanced NSCLC is currently ongoing. The sustained promising tumor activity of MRG003 justifies further anticipation.

### 6.2 BL-B01D1

BL-B01D1 is a bispecific ADC targeting EGFR and HER3, which induces cell cycle arrest in the S phase and subsequent apoptosis, leading to kill EGFR^+^ and/or HER3^+^ tumor cells. It is comprised of a bispecific antibody against EGFR/HER3 (SI-B001), a cathepsin B cleavable linker, and a novel topoisomerase I inhibitor (Ed-04), with a DAR of 8. Preclinical studies have shown that BL-B01D1 exhibits tumor suppressive effects in PDX models using human colorectal cancer cell lines and pancreatic cancer cell lines (72). In an FIH phase 1 clinical trial (NCT05194982), 76 NSCLC patients were evaluable for efficacy. The ORR in the subset of 34 NSCLC patients with EGFR mutant was observed to be 61.8% (CR: 15, PR: 6), while in the subgroup of 42 NSCLC patients with EGFR WT, the ORR was 40.5% (CR: 7, PR: 10). The most frequent TEAEs (>10%, all grade/≥ G3) were leukopenia (60%/30%), neutropenia (51%/34%), anemia (45%/15%), thrombocytopenia (44%/19%), alopecia (30%/0%), nausea (29%/<1%), vomiting (28%/0%), asthenia (21%/<1%), decreased appetite (22%/<1%), asthenia (21%/<1%), hypophagia (16%/0%), diarrhea (15%/2%), mouth ulceration (15%/<1%), rash (13%/0%). No cases of ILD were observed (73). In addition, there are ongoing clinical trials of BL-B01D1 as a single agent or combination therapy for metastatic or unresectable, advanced or metastatic NSCLC (NCT05983432, NCT05880706, and NCT05956587).

## 7 PTK7-targeted ADCs

The protein tyrosine kinase 7 (PTK7), also known as colon carcinoma kinase 4 (CCK4), is a receptor protein tyrosine kinase (74). Although PTK7 lacks catalytic activity within its kinase domain, it plays significant roles in canonical and non-canonical Wnt, as well as VEGF signaling (75). Additionally, PTK7 is highly expressed in diverse cancer cells, particularly NSCLC. The abnormal expression of PTK7, associated with multiple adverse prognoses, suggests its potential as a therapeutic target for NSCLC (76).

### 7.1 Cofetuzumab pelidotin

Cofetuzumab pelidotin is an ADC consisting of the anti-PTK7 monoclonal antibody cofetuzumab, conjugated to the microtubule inhibitor (Aur0101) through a cleavable linker, exhibiting a DAR of 4. Upon binding and internalization into PTK7-expressing cells, cofetuzumab pelidotin undergoes cleavage by intracellular proteases, leading to the release of the auristatin payload. This disrupts microtubules, induces G2-M phase cell cycle arrest, and triggers cell apoptosis, ultimately resulting in the death of cancer cells (77, 78). Preclinical studies have shown that treatment with cofetuzumab pelidotin leads to sustained regression of tumors in PDX models derived from patient samples, and it exhibits stronger anti-tumor activity compared to standard chemotherapy (77). A phase 1 clinical study (NCT02222922) involving patients with advanced solid tumors reported that neutropenia of grade 3 or above was experienced by 25% of participants. Two patients encountered dose-limiting toxicities, presenting as a grade 3 headache and fatigue. Antitumor activity was observed in treated NSCLC patients, where 6 out of 31 achieved PR, thus indicating an ORR of 19%. The RP2D was 2.8 mg/kg every 3 weeks. It is worth mentioning that patients with moderate or high expression levels of PTK7 were more responsive (79). Another ongoing phase 1 clinical trial (NCT04189614) is further investigating the effectiveness and safety of cofetuzumab pelidotin in patients with recurring PTK7^+^ NSCLC.

## 8 MSLN-targeted ADCs

Mesothelin (MSLN), a membrane-bound glycoprotein, is typically expressed at low levels in normal tissues. Conversely, there is observed overexpression of MSLN in various tumor cell types, including NSCLC (80). Overexpressed MSLN can stimulate resistance to apoptosis by activating NFκB, MAPK, and PI3K signaling pathways. This leads to enhanced cell proliferation, migration, and metastasis via the induction of MMP7 and MMP9 activation and expression (81–83). Hence, MSLN emerges as a promising potential therapeutic target for NSCLC.

### 8.1 Anetumab ravtansine

Anetumab ravtansine is an MSLN-targeted ADC, composed of a monoclonal antibody (MF-T) coupled with a microtubule inhibitor (DM4) via a reducible disulfide linker, exhibiting a DAR of 3.2 (84). The FIH study (NCT01439152) involving 148 patients with advanced or metastatic solid tumors, encompassing mesothelioma, ovarian, pancreatic, NSCLC, and breast cancers, SD was reported in 66 cases, including one with NSCLC. Additionally, PR was observed in 11 patients, and a CR was noted in one patient. The RP2D and schedule of anetumab ravtansine was determined as 6.5 mg/kg every three weeks or 2.2 mg/kg per week (85). Subsequently, two distinct clinical trials (NCT03455556, NCT02839681) were planned to determine the efficacy of anetumab ravtansine in advanced MSLN^+^ NSCLC patients. However, these trials were prematurely terminated due to slow patient recruitment and insufficient accrual.

## 9 B7-H3-targeted ADCs

B7-H3, also referred as CD276, is a transmembrane glycoprotein and belongs to the B7 ligand family. Although expression levels of B7-H3 are minimal in normal tissues, they markedly increase in a plethora of malignant tumors, including NSCLC, which correlates with poor prognosis (86). B7-H3 can instigate the migration and invasion of tumor cells, thereby escalating the progression of cancer (87). Furthermore, B7-H3 exerts immunosuppressive effects by promoting the infiltration of regulatory T cells within tumor tissues (88).

### 9.1 MGC018

MGC018 is an ADC composed of a B7-H3-targeted monoclonal antibody conjugated to a DNA-alkylating payload (duocarmycin) via a protease-cleavable linker with a DAR of 2.7. Its MOA includes payload-mediated DNA damage and bystander effects. In preclinical studies, antitumor activity was observed in PDX models. In addition, it showed good pharmacokinetics and safety in cynomolgus monkeys (89). In a phase 1/2 study (NCT03729596) involving 115 patients with advanced solid tumors, MGC018 demonstrated manageable safety and noticeable efficacy. Out of 16 evaluable patients with NSCLC, 4 patients achieved PR, with an ORR of 25%. The RP2D was determined as 3 mg/kg (90).

## 10 Tissue factor-targeted ADCs

Tissue factor, a transmembrane glycoprotein, plays a crucial role in the coagulation cascade and hemostasis under normal conditions. High expression of tissue factor is observed in various malignant tumors, including NSCLC. Its abnormal expression is strongly associated with tumor growth, enhanced metastasis, and poor prognosis (91).

### 10.1 Tisotumab vedotin (Tivdak)

Tisotumab vedotin is composed of the anti-tissue factor, monoclonal antibody (TF-011) conjugated to the payload MMAE via a protease-cleavable linker, with a DAR of 4.1 (92). Its main MOA *in vivo* is auristatin-mediated tumor cell killing. In addition, tisotumab vedotin has shown excellent anti-tumor activity in PDX models derived from solid cancer patients with different tissue factor expression levels, including models that showed tissue factor expression in only 25% to 50% of the tumor cells (92). In 2021, tisotumab vedotin has been approved by the FDA for treating patients with recurrent or metastatic cervical cancer. The FIH clinical trial of tisotumab vedotin (NCT02001623) was conducted in patients with advanced solid tumors. The RP2D was 2.0 mg/kg every three weeks. During the dose-expansion phase of the trial, a 13.3% ORR was recorded in 2 out of 15 patients diagnosed with NSCLC. The most frequent TEAEs (grade ≥ G3) included fatigue (10%), anemia (5%), abdominal pain (4%), and hypokalemia (4%). Notably, 69% of patients experienced epistaxis, potentially due to an impairment in tissue factor-mediated coagulation (93).

## 11 AXL-targeted ADCs

AXL is a transmembrane receptor tyrosine kinase and forms part of the TAM family (94). Upon activation, AXL stimulates several oncogenic signaling pathways such as PI3K and JAK/STAT (95). It plays an important role in promoting invasion and migration of tumor cells. Moreover, there is a correlation between AXL activation with resistance to EGFR-targeted treatments in the NSCLC (96).

### 11.1 Enapotamab vedotin

Enapotamab vedotin is an ADC composed of AXL-targeted monoclonal antibody (AXL-107) and a microtubule disrupting agent, MMAE, connected by a protease-cleavable linker (97). In preclinical studies, enapotamab vedotin exhibited significant single-agent activity in PDX NSCLC models expressing AXL, EGFR mutant, and EGFR inhibitor resistance (98). The FIH clinical trial of enapotamab vedotin (NCT02988817) was conducted with patients bearing relapsed or refractory solid tumors, recruiting a total of 47 patients, including 8 diagnosed with NSCLC. The preliminary data indicated that PR was observed in 3 patients, one of whom was diagnosed with NSCLC. The RP2D was determined as 2.2 mg/kg every three weeks (99). Phase 2a (expansion phase) of this study, included 26 patients with NSCLC, void of sensitizing EGFR mutant (EGFR WT) or ALK rearrangements (ALK^-^). The trial recorded an ORR of 19%, along with a DCR of 50%. 75% (9 of 12) evaluable fresh biopsies tested positive for AXL tumor cell staining (100).

## 12 NaPi2b-targeted ADCs

Sodium-dependent phosphate transport protein 2B (NaPi2b) is encoded by SLC34A2, which has been recognized to play a significant role in the regulation of tumor development (101). Studies have indicated elevated expression of NaPi2b in diverse cancers, especially notable in lung cancer patients exhibiting TTF1 positivity along with mutants in KRAS and EGFR (102). Such characteristics make it an appealing target for the development of ADC.

### 12.1 Lifastuzumab vedotin

Lifastuzumab vedotin is an ADC composed of anti-NaPi2b mAb (MNIB2126A) and a potent microtubule inhibitor (MMAE). Preclinical studies demonstrated that lifastuzumab vedotin exhibited significant anti-tumor efficacy in mouse models of ovarian cancer and the NSCLC PDX model, and it also showed acceptable safety in animal studies (103). In a phase 1a clinical trial (NCT01363947) involving patients with NSCLC and platinum-resistant ovarian cancer (PROC), 4 out of 51 NSCLC patients achieved PR, resulting in an ORR of 8%. The dose of 2.4 mg/kg was established as the RP2D. Lifastuzumab vedotin has limited efficacy in patients with NSCLC but is promising in patients with PROC, with an ORR of 46%. The most common AEs of any grade were fatigue (59%), nausea (49%), decreased appetite (37%), vomiting (32%), and peripheral sensory neuropathy (29%). The most common TEAEs (grade ≥ 3) were neutropenia (10%), anemia (3%), and pneumonia (3%) (104). Additionally, another phase 1 clinical trial (NCT01995188) is ongoing, also in patients with NSCLC and PROC.

### 12.2 XMT-1536

XMT-1536 is an ADC composed of a humanized anti-NaPi2B antibody conjugated to the payload AF-HPA with a high DAR of 10-15. AF-HPA is a cell-permeable anti-mitotic compound that slowly metabolizes into a highly low-permeable metabolite called auristatin F (AF) within the tumor, resulting in controlled bystander killing. The antitumor effect of XMT-1536 has been observed in preclinical studies using *in vivo* and *in vitro* models of adenocarcinoma, ovarian cancer, and lung cancer (105). Pharmacokinetic analysis showed approximately proportional increases in exposure in rats and monkeys. Systemic-free AF-HPA and AF concentrations were observed to be low in all animal species. The clinical activity of XMT-1536 is being evaluated in a phase 1/2 clinical trial (NCT03319628) involving patients with NaPi2b^+^ ovarian cancer and NSCLC. However, a separate clinical trial (NCT04396340) was terminated (106).

## 13 CEACAM5-targeted ADC

Carcinoembryonic antigen-related cell adhesion molecule 5 (CEACAM5), a cell surface glycoprotein, is typically expressed at low levels in the majority of normal tissues. However, its expression is significantly elevated in various tumors, notably those in the gastrointestinal tract, breast, and lung (107). Approximately 20% of patients diagnosed with NSCLC show overexpression of CEACAM5 (108). Thus, the deployment of ADCs targeting CEACAM5 could potentially exhibit promise for treating patients with NSCLC.

### 13.1 Tusamitamab ravtansine

Tusamitamab ravtansine is a CEACAM5-targeted ADC consisting of a humanized monoclonal antibody and a maytansinoid agent (DM4). It exerts anti-tumor activity by inhibiting tubulin polymerization through the action of DM4. Preclinical studies demonstrated the *in vitro* cytotoxicity and *in vivo* efficacy of tusamitamab ravtansine in a PDX model, as well as its safety profile in monkeys (109). A phase 2 clinical trial (NCT02187848) evaluated the efficacy and safety of tusamitamab ravtansine in patients with CEACAM5^+^ non-squamous NSCLC. This study enrolled a total of 92 individuals, of which 28 exhibited moderate IHC expression and 64 had high expression. The respective ORRs for moderate and high expression stood at 7.1% and 20.3%. Grade 3 or greater TEAEs occurred in 47.8% of patients, with 15.2% of them being assessed as drug-related (110). Additionally, tusamitamab ravtansine is currently under investigation in several ongoing clinical trials involving NSCLC patients, namely NCT04394624, NCT04524689, NCT05245071, and NCT04154956 (108, 111–113).

## 14 ROR2-targeted ADCs

Tyrosine-protein kinase transmembrane receptor (ROR2), a transmembrane protein receptor, is a member of the tyrosine kinase-like orphan receptor family. Despite lacking kinase function, it interacts with the non-canonical Wnt signalling (114). Research has demonstrated that ROR2 is significantly expressed in a range of malignant tumors, including NSCLC, and correlated with poor prognosis. It could potentially serve as a target for NSCLC treatment (115).

### 14.1 Ozuriftamab vedotin

Ozuriftamab vedotin is a novel ADC that consists of an anti-ROR2 monoclonal antibody conjugated to MMAE via a cleavable linker. Preclinical data suggest targeting ROR2 may result in antitumor activities in various tumor types, such as NSCLC. Multiple clinical trials for NSCLC are currently underway for ozuriftamab vedotin. The phase 2 clinical trial, NCT04681131, aims to assess the clinical efficacy of ozuriftamab vedotin as a single agent or in combination with nivolumab in patients with advanced solid tumors, including NSCLC (116). NCT03504488 is a phase 1/2 clinical trial evaluating the safety and efficacy of ozuriftamab vedotin in patients with NSCLC, TNBC, head and neck cancer, and melanoma.

## 15 ITGB6

Integrin subunit beta 6 (ITGB6) is an integrin protein heterodimer composed of an αv subunit and a β6 subunit. Normally, the expression of ITGB6 is low or absent in the epithelial cells of healthy tissues. However, its expression is increased during tissue repair and embryogenesis (117). Furthermore, studies have confirmed the overexpression of ITGB6 in various cancers, including NSCLC, which is associated with poor prognosis (118). Failure of ITGB6-based signaling mechanisms can result in abnormal cell division, adhesion, and migration, consequently contributing to tumorigenesis and metastasis (119).

### 15.1 SGN-B6A

SGN-B6A is an ITGB6-targeted ADC with MMAE as the payload. SGN-B6A exhibits anti-tumor activity through MMAE-mediated cytotoxicity, bystander effects, and immunogenic cell death. In preclinical studies, the antibody component of SGN-B6A specifically targets ITGB6, without binding to other members of the alpha-V family, and exhibits *in vivo* activity in models of NSCLC, pancreatic cancer, pharyngeal cancer, and bladder cancer (120). FIH phase 1 clinical trial (NCT04389632) is assessing the clinical activity of SGN-B6A in patients with advanced solid tumors. Out of the 27 patients with NSCLC who received treatment, 2 achieved CR and 7 achieved PR, resulting in an ORR of 33.3%. TEAEs occurred in 88.5% of patients: 50.7% were grade ≥ 3 (21.6% related), and 37.2% were serious (8.1% related). The most common TEAEs was fatigue (35.1%). Moderate neutropenia (8.1%) was the most frequently observed TEAEs grade ≥ 3 (121). Furthermore, another phase 3 clinical trial (NCT06012435) to evaluate the efficacy of SGN-B6A in patients with previously treated NSCLC is ongoing.

## 16 Conclusions and perspectives

ADCs, that are able to combine targeted therapy and cytotoxic chemotherapy, have demonstrated promising antitumor efficacy in preclinical and clinical trials, introducing a new treatment modality for advanced NSCLC patients (**Table 1**). Compared to conventional molecular targeted agents, ADCs offer an improved therapeutic index and have demonstrated more favorable clinical outcomes in certain NSCLC clinical trials (NCT04152499) (24, 141, 142). Despite the promising potential of ADCs in NSCLC therapy, the issue of drug resistance poses a significant challenge. For instance, telisotuzumab vedotin exhibits promising clinical activity for c-MET-positive NSCLC patients yet it provides limited clinical efficacy, with an ORR of merely 13.3%, in NSCLC patients with mutant EGFR (62, 64). EGFR mutations may be a one way through which cancer cells can escape the cytotoxic effects of telisotuzumab vedotin. Resistance to ADC may occur via multiple mechanisms: loss of internalization pathways preventing ADC internalization and transport; reduced lysosomal proteolysis or loss of lysosomal transporter function restraining linker cleavage and payload release within tumor cells; the upregulation of ATP-binding cassette transporter causing the direct transport and efflux of payload; the inactivation of pro-apoptotic proteins (Bak and Bax) or overexpression of anti-apoptotic proteins (Bcl-2 and Bcl-XL) leading to dysregulation of apoptotic pathways (142, 143). The mechanisms contributing resistance in NSCLC need further investigation.

**Table 1:** Efficacy of ADCs for NSCLC.

Target	Agent	Phase	Clinical trial ID	Patient	Efficacy	Adverse events	Reference
Trop2	Sacituzumab govitecan	1/2	NCT01631552	47	ORR: 19%	Grade 3 or higher AEs were experienced by 5% of the patients, including diarrhea (7%), nausea (7%), fatigue (6%), and neutropenia (28%)	(14)
		2	NCT06055465	–	–	–	–
		2	NCT05186974	61	Sacituzumab govitecan combined with pembrolizumab. ORR: 56%	The most common any-grade TEAEs were diarrhea (54%), anemia (48%), and asthenia (38%)	(16)
		3	NCT05089734	–	–	–	(122)
		3	NCT05609968	–	–	–	–
	Datopotamab deruxtecan	1	NCT03401385	180	ORR:24.4% (12/50, 4 mg/kg; 13/50, 6 mg/kg; 19/80, 8 mg/kg)	Grade ≥ 3 AEs in 47% of patients. TEAEs seen in ≥30% of patients included (all grade, grade ≥ 3) nausea (52%, 1%), stomatitis (48%, 2%), alopecia (39%, 0%), fatigue (32%, 1%), decreased neutrophil count/neutropenia (6%, 1%), diarrhea (16%, 0%), and ILD (11%, 2%)	(18)
		1	NCT04612751	–	–	–	–
		1	NCT04526691	132	Datopotamab deruxtecan plus pembrolizumab with or without chemotherapy (49%, n = 71; 38%, n = 61)	Grade 3 or higher TEAEs occurred in 53% and 76% of patients in the two groups who received doublet or combination chemotherapy, respectively	(20)
		1/2	NCT05460273	–	–	–	–
		2	NCT03944772	–	–	–	–
		2	NCT04940325	–	–	–	–
		2	NCT04484142	137 (56.9% had EGFR mutant)	ORR: 35.8% (EGFR mutant cohorts: 43.6%)	The most common grade ≥ 3 TEAEs were stomatitis (9.5%), anemia (5.8%), and increased amylase (5.8%)	(21)
		3	NCT04656652	299	ORR: 26.4%	The most common any grade TEAEs were stomatitis (49.2%) and nausea (37%)	(19)
		3	NCT05555732	–	–	–	(123)
		3	NCT05215340	–	–	–	(124)
	SKB264	1/2	NCT04152499	39	ORR: 44% (EGFR WT cohorts: 26%, TKI resistant EGFR mutant cohorts: 60%)	The most commonly observed grade ≥ 3 TEAEs, experienced by at least 5% of patients, consisted of d neutrophil count decreased (32.6%), anemia (30.2%), white blood cell count decreased (23.3%), stomatitis (9.3%), rash (7.0%), and lymphocyte count decreased (7.0%). Grade 4 TEAEs occurred only for neutropenia and white blood cell count decreased	(24)
		2	NCT05816252	–	–	–	–
		2	NCT05351788	–	–	–	–
		3	NCT05870319	–	–	–	–
	LCB-84	1/2	NCT05941507	–	–	–	(125)
	BL-M02D1	1/2	NCT05949619	–	–	–	–
HER2	Trastuzumab emtansine	2	NCT02675829	49	ORR: 51% (25/49)	TEAEs with total frequencies of greater than 10%, and no grade 4 or 5 AEs. Notable TEAEs included elevated levels of AST or ALT (63%), thrombocytopenia (31%), fatigue (16%), nausea (29%), infusion reactions (14%), anorexia (10%), and anemia (10%)	(28)
		Terminated	UMI000019446	15	ORR: 6.7% (1/15)	Grade 3 or 4 AEs included thrombocytopenia (40%) and hepatotoxicity (20%) without any TEAEs	(30)
		2	NCT02289833	49	ORR: 8% (IHC 2^+^: 0%, 4 IHC 3^+^: 20%)	Forty-five patients (92%) reported an AEs, and ten patients reported grade 3 AEs. Of AEs of particular interest in trastuzumab emtansine–treated patients, 1 event each of grade 3 thrombocytopenia and infusion-related reaction/hypersensitivity occurred	(31)
	Trastuzumab deruxtecan	2	NCT03505710	42 (HER2 mutant)	ORR: 61.9% (CR: 1, PR: 25)	All patients (42/42) had TEAEs; 64.3% were grade ≥ 3 (52.4% drug-related), including neutrophil count decreased (26.2%) and anemia (16.7%). There were 5 cases (11.9%) of drug-related ILD (all grade 2, no grade ≥ 3)	(34)
				49 (HER2 overexpression)	ORR: 24.5% (CR: 1, PR: 11)	All patients had ≥ 1TEAEs; the most common any-grade TEAEs were nausea (59.2%), decreased appetite (38.8%), and fatigue (32.7%). Grade ≥ 3 TEAEs were reported in 73.5% of patients (55.1% drug-related); the most common were decreased neutrophil count (20.4%) and fatigue (10.2%). There were 8 cases (16.3%) of drug-related ILD (grade 1, n = 2; grade 2, n = 3; grade 5, n = 3)	
	Trastuzumab botidotin	1/2	NCT03602079	–	–	–	(38)
		1	CTR20181301	–	–	–	(40)
	SHR-A1811	1/2	NCT04818333	50 (HER2 mutant)	ORR: 40%	All patients had TEAEs. 42% of patients experienced grade ≥ 3 TEAEs, with the most common ones being decreased neutrophil count (30%), white blood cell count decreased (20%), anemia (16%), and thrombocytopenia (12%). Nine patients (18%) had serious AEs deemed related to SHR-A1811. Treatment discontinuation due to AEs was reported in two patients. One death was reported to be treatment-related (ILD).	(43)
		1/2	NCT05482568	–	–	–	–
	XMT-1522	Terminated	NCT02952729	–	–	–	(46)
	Disitamab vedotin	2	NCT05847764	–	–	–	–
		2	NCT06003231	–	–	–	–
	Trastuzumab vedotin	2	NCT05141786	–	–	–	–
	ADCT-502	1	NCT03125200	–	–	–	–
HER3	Patritumab deruxtecan	1	NCT03260491	57	ORR: 39%	Grade ≥ 3 TEAEs were reported in 74% of patients. The most common grade ≥ 3 TEAEs were hematologic toxicities. Five patients were observed to have ILD, one of which was of grade 3	(53)
		1	NCT04676477	–	–	–	(126)
		2	NCT04619004	225 (HER2 mutant)	ORR: 29.8%	–	(54)
		2	NCT05865990	–	–	–	–
		3	NCT05338970	–	–	–	(55)
		–	NCT06099639	–	–	–	–
	YL-202	1	NCT05653752	–	–	–	–
c-MET	Telisotuzumab vedotin	1	NCT02099058	16	ORR: 18.8% (PR: 3)	–	(62)
		1		42	Telisotuzumab vedotin in combination with erlotinib. ORR: 30.6% (ORR of 32.1% in EGFR mutant cohorts)	Grade ≥ 3 TEAEs occurred in 13 patients (31%); the most frequently occurring were hypophosphatemia and peripheral sensory neuropathy (7% each). 3 of 42 patients (7%) reported ≥ 1 serious TEAEs: decreased appetite, dehydration, hemoptysis, peripheral neuropathy, and pneumonia (2% each)	(63)
		2	NCT03539536	88	ORR: 23% (EGFR WT cohort: 35.1%, EGFR mutant cohorts: 13.3%)	Grade 3 or higher AEs occurred in 50/113 (44%) patients, with most common (≥ 2%) being malignant neoplasm progression (6.2%), pneumonia (5.3%), hyponatremia (4.4%), anemia (2.7%), dyspnea (2.7%), fatigue (2.7%), increased GGT (2.7%), peripheral sensory neuropathy (2.7%), and pneumonitis (2.7%). Grade 5 TEAEs were sudden death, dyspnea, and pneumonitis (1 event each)	(64)
		2	NCT05513703	–	–	–	(66)
		3	NCT04928846	–	–	–	(67)
		Terminated	NCT03574753	–	ORR: 9%	3 fatal grade 5 pulmonary TEAEs (two instances of pneumonia and one of bronchopulmonary hemorrhage)	(65)
	RC108	1/2	NCT05821933	–	–	–	–
	REGN5093-M114	1/2	NCT04982224	–	–	–	(125)
	MYTX-011	1	NCT05652868	–	–	–	(127)
EGFR	MRG003	1	NCT04868344	–	–	–	(71)
		2	NCT04838548	–	–	–	–
	AVID100	1/2	NCT03094169	–	–	–	–
	CPO-301	1	NCT05948865	–	–	–	–
	AZD-9592	1	NCT05647122	–	–	–	(128)
EGFR, HER3	BL-B01D1	1	NCT05194982	76	ORR: 50% (EGFR WT cohorts: 40.5%, EGFR mutant cohorts: 61.8%)	The most common TEAEs (>10%, all grade/≥3) were leukopenia (60%/30%), neutropenia (51%/34%), anemia (45%/15%), thrombocytopenia (44%/19%), alopecia (30%/0%), nausea (29%/<1%), vomiting (28%/0%), asthenia (21%/<1%), decreased appetite (22%/<1%), asthenia (21%/<1%), hypophagia (16%/0%), diarrhea (15%/2%), mouth ulceration (15%/<1%), rash (13%/0%). No ILD was observed.	(73)
		1	NCT05983432	–	–	–	–
		2	NCT05880706	–	–	–	–
		2	NCT05956587	–	–	–	–
EGFR, MUC1	M-1231	1	NCT04695847	–	–	–	(129)
PTK7	Cofetuzumab pelidotin	1	NCT02222922	31	ORR: 19% (PR: 6)	The most common TEAEs were nausea, alopecia, fatigue, headache, neutropenia, and vomiting (45%–25%); 25% of patients had grade ≥ 3 neutropenia	(79)
		1	NCT04189614	–	–	–	–
MSLN	Anetumab ravtansine	1	NCT01439152	2	1 patient achieved SD	–	(85)
		Terminated	NCT03455556	–	–	–	–
		Terminated	NCT02839681	–	–	–	–
B7-H3	MGC018	1/2	NCT03729596	16	ORR: 25% (PR: 4)	–	(90)
B7-H4	SGN-B7H4V	1	NCT05194072	–	–	–	(130)
Tissue factor	Tisotumab vedotin	1/2	NCT02001623	15	ORR: 13.3%	The most frequent TEAEs (grade ≥ 3) included fatigue (10%), anemia (5%), abdominal pain (4%), and hypokalemia (4%)	(93)
	XB-002	1	NCT04925284	–	–	–	(131)
AXL	Enapotamab vedotin	1	NCT02988817	8	1 patient achieved PR	–	(99)
		2		26	ORR: 19%	Grade ≥ 3 TEAEs occurred in 12 patients, with the most common being gastrointestinal disorders in eight patients (constipation, n=1; colitis, diarrhea, nausea, vomiting, n=2 each; abdominal distension, n=1)	(100)
	Mecbotamab vedotin	2	NCT04681131		–	–	–
NaPi2b	Lifastuzumab vedotin	1	NCT01363947	51	ORR: 8% (PR: 4)	The most common AEs of any grade were fatigue (59%), nausea (49%), decreased appetite (37%), vomiting (32%), and peripheral sensory neuropathy (29%). The most common TEAEs (grade ≥ 3) were neutropenia (10%), anemia (3%), and pneumonia (3%).	(104)
		1	NCT01995188	–	–	–	–
	XMT-1536	1/2	NCT03319628	–	–	–	(106)
		Terminated	NCT04396340	–	–	–	–
	XMT-1592	1/2	NCT04396340	–	–	–	–
CEACAM5	Tusamitamab ravtansine	2	NCT02187848	92	ORR: 16% (PR: 15)	The most frequent TEAEs (all grades) were asthenia (38.0%), keratopathy/keratitis (38.0%), peripheral neuropathy (26.1%), dyspnea (23.9%), and diarrhea (22.8%). Grade ≥ 3 TEAEs occurred in 47.8% of patients and were assessed as drug-related in 15.2%	(110)
		2	NCT04394624	–	–	–	(113)
		2	NCT04524689	–	–	–	(112)
		2	NCT05245071	–	–	–	(111)
		3	NCT04154956	–	–	–	(108)
ROR1	Zilovertamab vedotin	2	NCT04504916		–	–	–
ROR2	Ozuriftamab vedotin	2	NCT04681131	–	–	–	(116)
		2	NCT03504488	–	–	–	–
ITGB6	SGN-B6A	1	NCT04389632	27	ORR: 33.3%, CR: 2, PR: 7	TEAEs occurred in 88.5% of patients: 50.7% were grade ≥ 3 (21.6% related), and 37.2% were serious (8.1% related). The most common TEAEs was fatigue (35.1%). Moderate neutropenia (8.1%) was the most frequently observed TEAEs grade ≥ 3	(121)
		3	NCT06012435	–	–	–	–
FOLR1	Farletuzumab ecteribulin	1	NCT03386942	4	ORR: 50% (PR: 2)	–	(132)
		2	NCT05577715	–	–	–	(133)
	PRO-1184	1/2	NCT05579366	–	–	–	(134)
TfR1	CX-2029	1/2	NCT03543813	9	ORR: 22.2% (PR: 2)	–	(135)
CD25	Camidanlumab tesirine	1	NCT03621982	–	–	–	–
CD166	CX-2009	1/2	NCT03149549	–	–	–	(136)
CD228	SGN-CD228A	1	NCT04042480	–	–	–	(137)
LIV-1	Ladiratuzumab vedotin	2	NCT04032704	–	–	–	–
EphA2	MM-310	1	NCT03076372	–	–	–	(138)
ASCT2	MEDI-7247	1	NCT03811652	–	–	–	–
5T4	PF-06263507	1	NCT01891669	–	–	–	–
HER2, TLR8	SBT6050	1	NCT04460456	–	–	–	–
		1/2	NCT05091528	–	–	–	(139)
HER2, STING	XMT-2056	1	NCT05514717	–	–	–	–
CCR2, STING	TAK-500	1	NCT05070247	–	–	–	(140)

**Figure 1:**
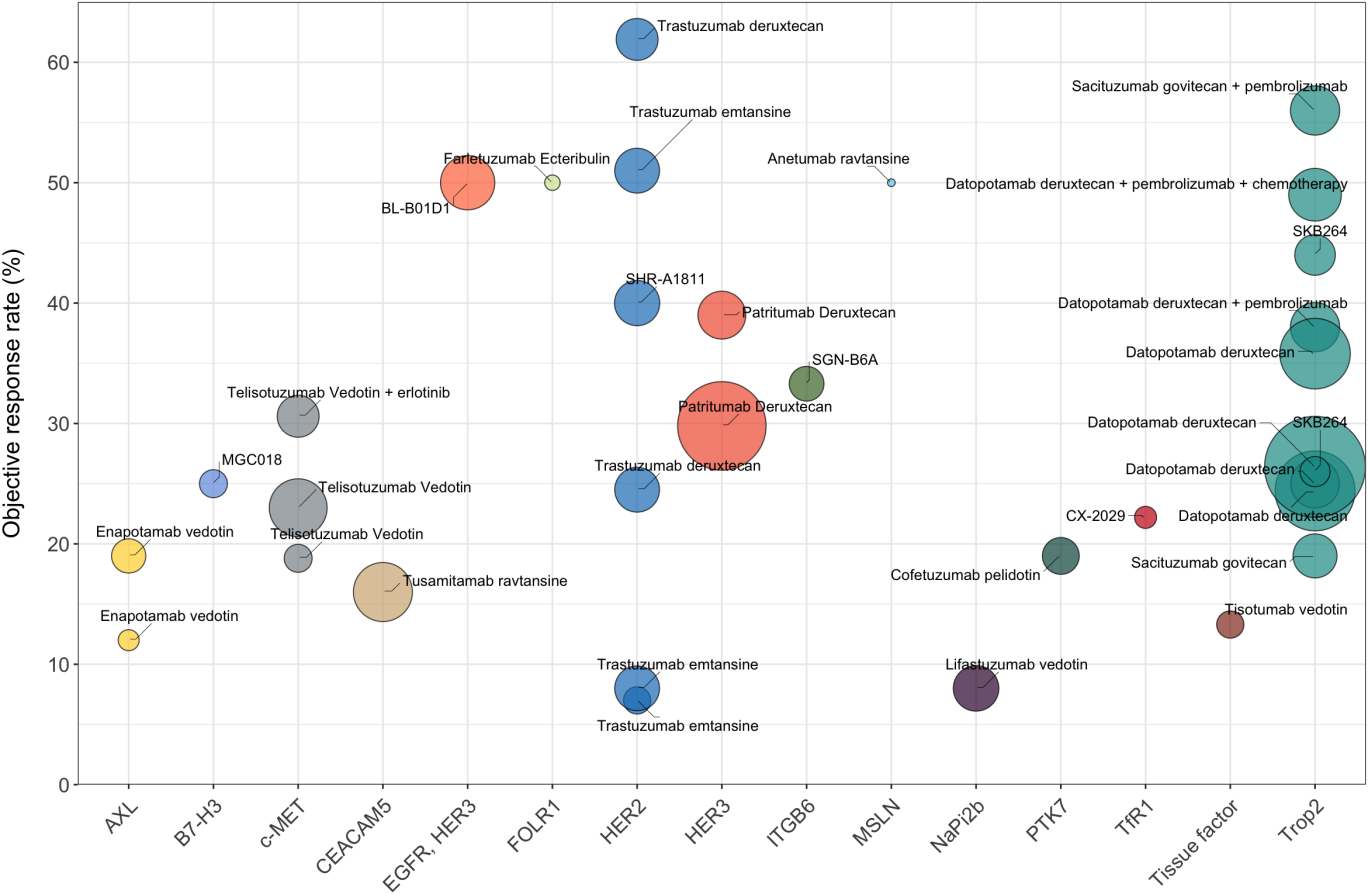
The efficacy data (ORR) of different ADCs for the treatment of NSCLC (limited to ADCs with published clinical results). Trastuzumab deruxtecan, as a monotherapy, has the highest ORR of 61.9%. Sacituzumab govitecan combined with pembrolizumab demonstrates an ORR of 56%. Bispecific ADC serves as another promising therapeutic modality, with BL-B0D1 demonstrating an ORR of 50% in NSCLC patients. The fill color of the circle represents different targets for NSCLC. The sizes of the circles represent the number of evaluable patients with NSCLC.

Combinations with other antitumor agents or use of multi-specific ADCs targeting different antigens may be alternative approaches to overcome resistance in NSCLC treatments (**Figure 1**). For example, the combination of telisotuzumab vedotin with erlotinib resulted in an ORR of 32.1% among NSCLC patients with EGFR mutation, significantly surpassing the ORR observed with telisotuzumab vedotin monotherapy (63). The combination of ADCs with immune checkpoint inhibitors (ICIs) has also exhibited powerful tumor-killing activity. For instance, the combination of sacituzumab govitecan and pembrolizumab showed promising clinical activity as a first-line treatment for metastatic NSCLC (**Figure 1**). The next aspect to consider for future research is how to optimize the risk-benefit profiles of ADCs in NSCLC patients. Some target antigens for NSCLC ADCs, such as Trop2, are widely expressed in normal tissues, which potentially leads to excessive exposure of normal tissues and can result in unmanageable toxicity. Therefore, the ongoing quest to discover more effective and safer ADCs that have advantageous tumor-specificity in NSCLC remains a critical focus for future research.

## Author contributions

XL: Conceptualization, Funding acquisition, Resources, Writing – original draft. JD: Writing – original draft, Writing – review & editing. RZ: Writing – review & editing. JXi: Writing – review & editing. YW: Writing – review & editing. WC: Writing – review & editing. BL: Writing – review & editing. DX: Writing – review & editing. JXu: Resources, Writing – review & editing. MZ: Writing – review & editing, Conceptualization.

## Glossary

**Table:**

ADCs	antibody-drug conjugates
AEs	adverse events
c-MET	mesenchymal-epithelial transition factor
CR	complete response
DCR	disease control rate
EGFR	epidermal growth factor receptor
FIH	first-in-human
FDA	Food and Drug Administration
HER2	human epidermal growth factor receptor 2
HER3	human epidermal growth factor receptor 3
ICI	immune checkpoint inhibitor
ITGB6	Integrin subunit beta 6
ILD	interstitial lung disease
LSCD	limbal stem cell deficiency
MOAs	mechanism of action
mDOR	median duration of response
mOS	median overall survival
PFS	median progression-free survival
MSLN	mesothelin
mTNBC	metastatic triple-negative breast cancer
MMAE	monomethyl auristatin E
NCCN	National Comprehensive Cancer Network
NSCLC	non-small cell lung cancer
NaPi2b	sodium-dependent phosphate transport protein 2B
ORR	objective response rate
PR	partial response
PDX	patient-derived xenografts
PROC	platinum-resistant ovarian cancer
PTK7	protein tyrosine kinase 7
RP2D	recommended phase 2 dose
SD	stable disease
TEAEs	treatment emergent adverse events
Trop2	trophoblast cell surface antigen 2
TKIs	tyrosine kinase inhibitors
ROR2	Tyrosine-protein kinase transmembrane receptor
